# The New Health Act

**Published:** 1921-04-02

**Authors:** 


### THE HOSPITAL WORLD IN NEW ZEALAND.

### The New Health Act.

The new Health Act for New Zealand came into force on January 1, 1921. The member of the Executive Council holding office as the Minister of Public Health becomes the Minister of Health, and the Department of State is called the Department of Health. It comprises the following divisions: Public Hygiene; Hospitals; Nursing; School Hygiene; Dental Hygiene; Child Welfare; Maori Hygiene; and consists of the following *personnel*:

(*a*) The Minister of Health, who shall have the general administration of the Department; (*b*) the Board of Health, constituted by the Act; (*c*) a chief administrative officer, to be called the Director-General of Health, who shall be a medical practitioner, with specific qualifications in sanitary science; (*d*) a Deputy Director-General of Health, who shall be a medical practitioner with special qualifications in sanitary science; (*e*) Directors of the several Divisions; (*f*) such number of medical officers of health as may be required, who shall be medical practitioners with special qualifications in sanitary science; (*g*) such number of inspectors of health as may from time to time be required, who shall be holders of approved certificates in sanitation; (*h*) such number of medical practitioners, nurses, and other professional officers as may from time to time be required; (*i*) an officer to be called the Secretary of the Department of Health; and (*j*) such other officers, whether permanent or temporary, as may from time to time be found necessary.

All officers holding office as officers of the Public Health Department under "The Public Health Act, 1908," are deemed to be officers permanently appointed to hold corresponding positions in the Department of Health under the new Act.

### Constitution of the Board of Health.

The Board of Health consists of—(*a*) The Minister of Health, who shall be Chairman of the Board; (*b*) the Director-General of Health; (*c*) a medical practitioner, to be appointed on the recommendation of the Minister; (*d*) a medical practitioner, being a member of the Medical Board constituted under the Medical Practitioners Act, 1914, to be appointed on the recommendation of the New Zealand Branch of the British Medical Association; (*e*) a medical practitioner, being a member of the Faculty of Medicine in the University of Otago, to be appointed on the recommendation of the Minister; (*f*) a person (not being a medical practitioner), to be appointed on the recommendation of the New Zealand Municipal Association; (*g*) a person (not being a medical practitioner), to be appointed on the recommendation of the New Zealand Counties Association; (*h*) a member of a recognised Association of Civil Engineers, to be appointed on the recommendation of the Minister; (*i*) a person, being the Chairman of a Hospital and Charitable Aid Board, to be appointed on the recommendation of the Minister; and (*j*) two other persons (one of whom shall be a woman), to be appointed on the recommendation of the Minister.

### Functions of Department and Board.

The functions of the Department of Health are to advise local authorities in matters relating to public health in so far as those local authorities are charged with the care of the public health; the prevention, limitation, and suppression of infectious and other diseases; to promote and carry out researches and investigations in relation to matters concerning the public health, and the prevention or treatment of disease; to publish reports, information, and advice concerning the public health; the organisation and control of medical, dental, and nursing services, so far as such services are paid for out of public moneys, not being services in connection with any institution established under the Mental Defectives Act, 1914; generally to take all such steps as may be desirable to secure the prevention, effective carrying out, and co-ordination of measures conducive to the public health; the functions of the Board of Health are to hold such inquiries, to give all such decisions, awards, determinations, recommendations, and consents, and to do all such other acts as may in its opinion be necessary for the effective administration of the Health Act.

### THE WELFARE OF HOSPITAL STAFFS.

Under an amending Act entitled "The Hospitals and Charitable Institutions Act Amendment Act, 1920," a Hospital Board may pay to the Chairman of the Board by way of remuneration for his services such sum as it thinks fit, not exceeding £100, in respect of any financial year. Provision is authorised, subject to the approval of the Minister, for the establishment of bursaries for students of nursing or massage; and of pensions on retirement for any officers and servants of the Board provided that no such pension shall exceed the rate of £2 a week, or be payable to any person who has had less than ten years' continuous employment in the service of the Board.

The Governor-General is empowered to authorise the making of regulations for the protection of the interests and the promotion of the welfare of nurses engaged in public hospitals. Regulations may relate to the accommodation to be provided for nurses, the leave of absence from duty to be from time to time allowed, the working conditions, and generally such other matters as the Governor-General thinks fit.

The Government has held over the proposed schedule of proportional subsidy. A Commission of Inquiry is to be set up to take evidence of Hospital Boards, Departmental officers, and actuaries for the purpose of devising a satisfactory and equitable system of subsidy.

"The Nurses' Registration Amendment Act, 1920," provides for registration at the age of twenty-one, which means that a probationer may enter for her three years' course of training at the age of eighteen years.

